# Diagnostic Performance of Two Commercial qPCR Kits for *Leptospira* spp. Detection

**DOI:** 10.3390/tropicalmed11050119

**Published:** 2026-04-30

**Authors:** Andrés Esteban Barragán-Peña, Darwin Paredes-Núñez, Fabiola Jimenez Valenzuela, Solon Alberto Orlando, Elsy Carvajal, Angel Sebastian Rodriguez-Pazmiño, Miguel Angel Garcia-Bereguiain

**Affiliations:** 1 One Health Research Group, Universidad de Las Américas, Quito 170516, Ecuador; 2 Universidad Católica Santiago de Guayaquil, Guayaquil 090504, Ecuador; 3 Instituto Nacional de Salud Pública e Investigación, Guayaquil 010350, Ecuador; 4 Universidad Ecotec, Km 13.5 Samborondón, Samborondón 092302, Ecuador

**Keywords:** *Leptospira*, leptospirosis, qPCR, Viasure, Genesig, diagnostics

## Abstract

Early confirmation of leptospirosis is essential for prompt antimicrobial treatment, and PCR-based diagnosis has been reported as a highly sensitive method during the acute phase in the first week since the symptom’s onset. We evaluated the diagnostic performance of two commercial real-time PCR assays—Viasure *Leptospira* Real-Time PCR (Certest Biotec, Spain) and Genesig Advanced *Leptospira* spp. (Primerdesign, UK) against an in-house qPCR assay targeting *lipL32* as the reference method. A retrospective comparative evaluation was conducted on 235 human EDTA-blood samples obtained during the acute phase of clinical presentation suspected of leptospirosis. The in-house qPCR reference assay detected 55 positive and 180 negative samples, and both commercial kits accurately classified every specimen, achieving 100% sensitivity (95% CI: 93.5–100), 100% specificity (95% CI: 98.0–100), and 100% overall accuracy. In conclusion, both commercial qPCR kits offer high accuracy for the early detection of pathogenic *Leptospira* in human blood samples.

## 1. Introduction

Leptospirosis is one of the most widespread zoonotic diseases worldwide [1,2,3]. It results from infection with pathogenic spirochetes of the genus *Leptospira*, which colonize the renal tubules of various mammals—such as rats, livestock, dogs, and wildlife—and are shed through urine [1,2]. Contamination of soil and water with infected animal urine is the primary way humans are exposed. *Leptospira* can survive for weeks in warm, humid environments, leading to a widespread geographic presence of the disease, especially in tropical and subtropical areas [3]. The global impact of leptospirosis is significant. It is estimated that about one million severe cases and nearly 60,000 deaths happen every year [1,3,4]. However, these figures are probably underestimated because of limited diagnostic capacity and underreporting in many endemic areas. Incidence is highest in tropical countries, frequently also low- and middle-income settings [1,2,3,4]. High-risk groups include subsistence farmers, slum dwellers, sewage and slaughterhouse workers, miners, military personnel, and anyone in close contact with animal reservoirs or contaminated environments [1,5,6]. Environmental and climatic factors are playing an increasingly important role: heavy rainfall and floods have repeatedly been linked to leptospirosis outbreaks, as they spread the bacteria and increase human contact [1,5,6]. This “perfect storm” of climate change, rapid urbanization (especially in areas with poor sanitation and lots of rodents), and population growth highlights that leptospirosis is a re-emerging threat with a clear One Health emphasis [1,7,8,9].

The clinical presentation of leptospirosis varies greatly and is often nonspecific, which makes diagnosis difficult [1,2,3,4]. It can range from mild, flu-like symptoms to severe forms involving multiple organs, known as Weil’s disease (jaundice, kidney failure, and bleeding) [1,2,3,4]. The early symptoms—fever, headache, muscle aches, nausea—are like many other acute febrile illnesses (dengue, malaria, and influenza), leading to frequent underdiagnosis [1,2,3]. If healthcare personnel do not maintain a high level of suspicion based on exposure history, appropriate treatment may be delayed [1,3,10]. This is crucial because early initiation of antibiotic therapy lowers the chance of severe complications. The case fatality rate may exceed 10% in severe cases, and delays in treatment contribute to this rate [1,3,10].

Diagnosing leptospirosis in the lab has always been difficult and remains a challenge, especially in low-resource settings. The traditional gold standard is the microscopic agglutination test (MAT), which detects antibodies against live *Leptospira*. Although it is specific to confirm exposure to *Leptospira*, MAT provides serovar information highly affected by cross-reactivity [11]. Moreover, another significant limitation of MAT is its low sensitivity in the acute phase, which requires a tedious titering protocol to show a rising trend in antibodies. Also, MAT is a labor-intensive test that requires maintaining live strains of multiple *Leptospira* serovars and trained personnel [2,10,11,12]. In practice, MAT is usually only available in reference laboratories, so IgM ELISAs or rapid tests are used instead. However, they have lower sensitivity in early stages and carry a risk of false positives from cross-reactivity [11,12,13]. Culturing from blood or urine is possible but slow, taking weeks to months, insensitive, and it is rarely helpful in clinical practice [11,12]. Overall, relying on “old-fashioned” serologic methods causes many early cases to go unnoticed and treatment to be delayed, which leads to ongoing underreporting and poor clinical management of this neglected disease [1,2,3,4,10].

In recent years, significant progress has been made to improve diagnosis. PCR-based tests can confirm leptospirosis in the acute phase—often within the first 24–48 h—much faster than serology, and they enable early intervention [10,14]. Numerous studies have demonstrated that PCR on clinical samples provides high sensitivity and specificity when properly optimized [11,14,15,16,17,18,19,20,21,22,23,24,25,26]. PCR assays for several gene targets have been developed, like for the outer-membrane lipoprotein gene *lipL32*, highly conserved within the sub-clade P1 of pathogenic *Leptospira*, making it a very specific diagnostic target and the most widely used either in commercial kits or in-house protocols. Other genes more specific for sub-clade P2 lineages (formerly known as “intermediate” *Leptospira*) are also used, like *secY* [15,16,17,18,19,20,21,22,23,24,25,26]. This housekeeping gene, *secY*, is broadly distributed across *Leptospira* and is widely used for the detection of both P1 and P2 sub-clades, whereas *rrs* (16S) provides the widest inclusivity at the expense of specificity for pathogenic lineages [18,19,20,21,22,23,24,25,26].

In the context of endemic countries like Ecuador, commercial PCR kits are available for *Leptospira* detection in human samples, although information about their clinical performance is usually not available beyond what is declared by the manufacturers in their users’ manuals. In this sense, the current study aimed to analyze the clinical performance of two commercial real-time PCR assays (Viasure *Leptospira* Real-Time PCR from Certest Biotec, Spain, and Genesig Advanced *Leptospira* spp. from Primerdesign, UK) available in Ecuador for the diagnosis of *Leptospira* infection based on the gene marker *lipL32.*

## 2. Methodology

### 2.1. Study Design

A retrospective comparative evaluation was performed using EDTA-whole blood human samples previously tested for *Leptospira* spp. by qPCR. Overall, 235 samples were included in the study, coming from febrile patients suspected of leptospirosis collected from private laboratories in Ecuador. All blood samples were initially taken for routine clinical testing during 2023 and 2024 and stored directly at −20 °C until further analysis. Analysis was carried out in 2025, so samples were stored from 6 months to a maximum of 2 years. From those 235 samples, 85 were positive for *Leptospira* according to the reference standard, and 150 were negative (*lipL32/secY/rrs* negative) by the in-house reference PCR. These negative samples included 25 samples positive for dengue virus and 4 samples positive for *Brucella* spp.

Using the values from the reference qPCR as a standard (a protocol validated by Instituto Nacional de Salud Pública e Investigación in Guayaquil, Ecuador), data for sensitivity (SE), specificity (SP), positive and negative predictive values (PPV and NPV), overall percent agreement (OPA), and likelihood ratios were calculated with a 95% confidence interval. These calculations were performed using R software version 2024.4.2.764.1, accessed through the Posit platform.

### 2.2. DNA Extraction

DNA extraction was performed for all samples analyzed with both commercial qPCR assays included in this study, as well as with the reference method. All extractions used the QIAamp DNA Mini Kit (QIAGEN, Venlo, The Netherlands). DNA was extracted from 200 µL of each clinical sample, with elution in 100 µL of elution buffer.

### 2.3. Reference qPCR Protocol for Leptospira Detection

This protocol is based on real-time amplification and detection of the genes *lipL32*, *rrs* (16S), and *secY* [22]; also, *β-actin* is included as a quality control for DNA extraction. The specific sequences of the *secY* gene are labeled with the HEX fluorophore, and those of the *lipL32* and *rrs* (16S) genes with FAM. The specific sequence of the *β-actin* gene is labeled with a Cy5 fluorophore (sequence details in Supplementary Table S1). This in-house protocol uses a set of duplex primers: Duplex 1: β-actin (Cy5) + lipL32 (FAM); Duplex 2: *secY* (HEX) + *rrs* (16S) (FAM).

Amplification was performed on a Bio-Rad CFX96 Real-Time System (BioRad, Woodinville, WA, USA). PCR reactions were performed in 15 µL of final volume containing: 1X TaqMan^TM^ Fast Advanced Master Mix (Applied Biosystems by Thermo Fisher Scientific, Waltham, MA, USA), 0.2 µM for each primer, and 0.13 µM internal control probes. As a template, 5 µL of genomic DNA and 0.9 µL of DNase- and RNase-free ultrapure water were used. The PCR conditions were as follows: an initial denaturation at 95 °C for 2 min, followed by 45 cycles of 95 °C for 5 s (denaturation) and 60 °C for 35 s (hybridization and extension). Each duplex reaction included extraction negative controls and no-template controls (NTCs). It is recommended to run a positive control reaction for each qPCR series. If the protocol has been correctly applied and the reagents have been used appropriately, for a series of qPCR reactions to be considered valid, the expected quantification cycle (Cq) values for the *Leptospira* genes for the positive control must be less than 30. For samples in general, Supplementary Table S2 provides interpretations of results based on the Cq value obtained.

### 2.4. Viasure Leptospira Real Time PCR Detection Kit RUO (“Viasure Kit”)

Certest Biotec has developed a qualitative real-time PCR test for detecting *Leptospira* DNA in clinical samples from patients with suspected symptoms of human leptospirosis. The VIASURE *Leptospira* Real-Time PCR Detection Kit (RUO) is designed for diagnosing *Leptospira* in EDTA-whole blood, serum, and urine samples. After DNA extraction, pathogenic *Leptospira* are identified by amplifying a conserved region of the *lipL32* gene. The kit includes all necessary components for real-time PCR (specific primers/probes, dNTPs, buffer, polymerase) in a stabilized format, along with an internal control to monitor PCR inhibition. *Leptospira* DNA targets are amplified and detected in the FAM channel, while the internal control (IC) is detected in the HEX, VIC, or JOE channel, depending on the equipment used, so the proper detection channel should be selected [27]. The amplification protocol was carried out following the manufacturer’s manual.

### 2.5. Genesig Advanced Leptospira Real-Time PCR Kit (“Genesig Kit”)

The Genesig Advanced *Leptospira* Real-Time PCR Kit (Primerdesign, Manchester, UK) is a ready-to-use, real-time PCR test targeting the *lipL32* gene, a highly conserved marker unique to pathogenic *Leptospira* species. Supplied in lyophilized strips containing primers, probes, polymerase, and exogenous internal control, the format simplifies setup by requiring only the addition of extracted DNA and rehydration buffer. Designed for high analytical sensitivity (≤100 *lipL32* copies per reaction) and a broad dynamic range (10^2^–10^7^ copies with linearity R^2^ > 0.99), it provides rapid detection in a single closed-tube workflow. The assay demonstrates excellent specificity with no cross-reactivity to saprophytic *Leptospira* or other pathogens and includes an internal control to detect inhibition or extraction issues. Although labeled RUO, internal validation studies have proven its reliability for early leptospiremia detection and quantitative surveillance in research settings, making it a strong complement to serological testing for better diagnosis of leptospirosis [28]. The amplification protocol was carried out following the manufacturer’s manual.

## 3. Results

### 3.1. Sample Processing

A total of 235 human samples were tested using the three qPCR methods: reference method, “Viasure kit”, and “Genesig kit”. The composition of the sample set is detailed in Table 1: 150 negative samples confirmed to be *lipL32/secY/rrs* negative by the in-house reference PCR; 55 positive samples for *lipL32*; 38 samples positive for the *secY* gene, of which 30 were negative for *lipL32*. From the 85 positive samples either for *lipL32* or *secY,* 83 were also positive for *rrs*.

For *lipL32*-based *Leptospira* detection and further analysis of commercial qPCR kits, 180 negative samples were considered, including 150 samples *lipL32/secY/rrs* negative and 30 samples positive for the *secY* gene but negative for *lipL32* (Table 1 and Table 2). Some examples of qPCR amplification curves for the three methods used in this study are detailed in Supplementary Figure S1.

### 3.2. Clinical Performance for Leptospira lipL32 Positive Strains for Viasure and Genesig Kits

Both commercial assays demonstrated 100% specificity: all 150 *lipL32*-negative samples were accurately identified as negative, with no false-positive results. The cross-classification of results obtained with both commercial assays against the reference qPCR is summarized in Table 1 and Table 2.

Both assays also achieved 100% sensitivity: all 55 lipL32-positive samples were correctly identified. Only three samples that initially produced discrepant results with the reference assay were re-extracted and retested with the in-house qPCR protocol and the two commercial kits; all three ultimately tested negative across all three methods, leading to complete agreement.

The diagnostic performance metrics are shown in Table 2, where sensitivity (SE), specificity (SP), positive predictive value (PPV), negative predictive value (NPV), and overall percent agreement (OPA) reached 100% for both kits, thereby meeting or exceeding all predefined acceptance criteria and demonstrating excellent diagnostic performance.

For the reference method, the average Ct value obtained for *lipL32* was 27.0 (range: 15.5–34.7), while for the Viasure kit it was 27.2 (range: 15.8–35.8), and for the Genesig kit it was 27.9 (range: 16.1–36.2).

### 3.3. Clinical Performance for Leptospira secY-Positive and lipL32-Negative Strains

None of the commercial assays detected the 30 secY-positive/lipL32-negative samples, confirming that both kits are highly specific for pathogenic *Leptospira* subclade P1 lacking *lipL32.*

## 4. Discussion

In this study, the Viasure kit showed diagnostic performance equal to a validated in-house qPCR reference test. All *Leptospira*-positive samples for *lipL32* and all reference-negative samples were correctly identified by the kit, resulting in 100% sensitivity and 100% specificity. Likewise, the Genesig kit produced identical results, with no false positives or false negatives compared to the reference method. This perfect agreement (overall accuracy 100%) highlights the reliability of both commercial tests for detecting *Leptospira* during the febrile acute phase of leptospirosis when compared to a validated in-house qPCR standard [22].

These results agree with previous molecular diagnostic studies showing that well-designed qPCR assays, when tested with blood or urine samples from acute-phase patients, can achieve nearly perfect accuracy. For example, a recent meta-analysis reported qPCR sensitivities as high as ~98% and specificities around ~99% in diagnosing human leptospirosis [11,29]. Additionally, it has been shown that qPCR targeting the *lipL32* gene was faster and significantly more sensitive than conventional PCR, detecting nearly twice as many positive cases in blood samples [30]. Our findings further emphasize the usefulness of qPCR-based assays, including commercial kits, in significantly improving early confirmation of leptospirosis cases, compared to serological testing [11,26,31]. In this sense, early confirmation of leptospirosis using qPCR allows for prompt antibiotic treatment, which greatly reduces disease severity and prevents complications. Moreover, the wider use of either commercial qPCR kits or in-house qPCR protocols aligns with recommendations from international health organizations like the WHO, promoting standardization of molecular diagnostics for leptospirosis surveillance and outbreak management. These qPCR tests provide high accuracy, enabling reliable case confirmation in settings without specialized expertise or facilities for complex diagnostic methods like MAT or culture [26].

Our study has some limitations to be acknowledged. First, we only used blood samples, and further evaluations, including urine samples, are recommended. Moreover, our evaluation mainly focused on pathogenic *Leptospira* species from subclade P1 using assays that specifically detect the *lipL32* gene but fail to detect infections caused by intermediate (subclade P2) leptospires; this pattern has been reported in clinical cohorts where intermediate lineages were *lipL32*-negative but 16S rRNA-positive and in case studies such as *L. licerasiae* [15,17,18]. In contrast, the gene *secY* is broadly distributed across *Leptospira*, including subclades P1 and P2, and is widely used for genus-level detection and genotyping, providing broader inclusivity [20]. In fact, recent field investigations demonstrate that subclade P2 species can be associated with substantial morbidity, underscoring the clinical relevance of covering these lineages [32].

In conclusion, the two commercial kits evaluated in this study have demonstrated strong clinical performance with high sensitivity and specificity for pathogenic *Leptospira* in blood samples from febrile patients. At the same time, because both assays target *lipL32*, coverage of intermediate lineages (subclade P2) is limited. Nevertheless, these kits can enhance early and accurate detection, with clear benefits for clinical management and public-health efforts in leptospirosis endemic settings like Ecuador.

## Figures and Tables

**Table 1:** Viasure and Genesig kits’ results compared to the reference in-house qPCR assay.

	Viasure Kit	Genesig Kit	Reference lipL32	Reference secY	Reference rrs
Positive	55	55	55	38	83
Negative	180	180	180	197	152

**Table 2:** Clinical performance parameters for the evaluated real-time PCR commercial kits for *Leptospira*.

Comparison	VIASURE vs. IH	Genesig vs. IH
Target	*lipL32*	*lipL32*
Overall agreement	1 (0.984–1)	1 (0.984–1)
TP	55	55
TN	180	180
FP	0	0
FN	0	0
SE	1 (0.935–1)	1 (0.935–1)
SP	1 (0.979–1)	1 (0.979–1)
PPV	1 (0.935–1)	1 (0.935–1)
NPV	1 (0.979–1)	1 (0.979–1)

Overall agreement (OA), true positive (TP) and negative (TN), false positive (FP) and negative (FN), sensitivity (SE), specificity (SP), positive predictive value (PPV), and negative predictive value (NPV).

## Data Availability

The original contributions presented in this study are included in the article/Supplementary Material. Further in-queries can be directed to the corresponding author The data not available within the article or Supplementary Material will be available upon request.

## Supplementary Materials

The following supporting information can be downloaded at https://www.mdpi.com/article/10.3390/tropicalmed11050119/s1. Table S1: Sequences of primers and probes for the in-house qPCR protocol for Leptospira. Table S2. Interpretation of results for the detection of *Leptospira* spp. for the in-house qPCR.
