# Supplementary material for: Diagnostic Performance of Two Commercial qPCR Kits for Leptospira spp. Detection
Source: Trop Med Infect Dis. 2026 Apr 30;11(5):119. doi: 10.3390/tropicalmed11050119 (PMC13211705; doi:10.3390/tropicalmed11050119)
Supplement: Supplementary file 1 [file tropicalmed-11-00119-s001.zip › tropicalmed-4171538-supplementary.pdf]

Supplementary Table S1. Sequences of primers and probes for the in-house qPCR protocol for *Leptospira*

Duplex 1		
**Gene**	**Primer and Probe**	**Sequence**
β-actin	F_actin (fw)	5′-GGCTCYATYCTGGCCTC-3′
	R_actin (rv)	5′-GCAYTTGCGGTGSACRATG-3′
	P_actin (p)	5′-CY5TACTCCTGCTTGCTGATCCACATCBHQ2-3′
*lipL*32	F_lip32 (fw)	5′-AAGCATTACCGCTTGTGGTG-3′
	R_lip32 (rv)	5′-GAACTCCCATTTCAGCGATT-3′
	taq-189P (p)	5′-FAMAAAGCCAGGACAAGCGCCGBHQ1-3′
**Duplex 2**		
**Gene**	**Primer and Probe**	**Sequence**
secY	F_Lint2 (fw)	5′-CTTGAGCCTGCGCGTTAYC-3′
	R_Lint2 (rv)	5′-CCGATAATTCCAGCGAAGATC-3′
	TaqLint2 (p)	5′-HEXCTCATTTGGTTAGGAGAACAGATCABHQ1-3′
rrs (16S)	F_Lept (fw)	5′-CCCGCGTCCGATTAG-3′
	R_Lept (rv)	5′-TCCATTGTGGCCGRA/GACAC-3′
	P_Lept (p)	5′-FAMCTCACCAAGGCGACGATCGGTAGCBHQ1-3′

fw, forward; rv, reverse; p, probe; BHQ1, black hole quencher 1; BHQ2, black hole quencher 2

Supplementary Table S2. Interpretation of results for the detection of *Leptospira* spp for the in-house qPCR.

***sec*Y (HEX)**	***lipL*32 (FAM)**	***rrs* (16S) (FAM)**	***β*-actin (Cy5)**	**Result**
Cq ≤ 38	No amplification	No amplification	Irrelevant	*Leptospira* positive
No amplification	Cq ≤ 38	No amplification	Irrelevant	*Leptospira* positive
No amplification	No amplification	Cq ≤ 38	Irrelevant	*Leptospira* positive

No amplification	No amplification	No amplification	Cq ≤ 40	Negative
No amplification	No amplification	No amplification	No amplification	Invalid (repeat DNA extraction)

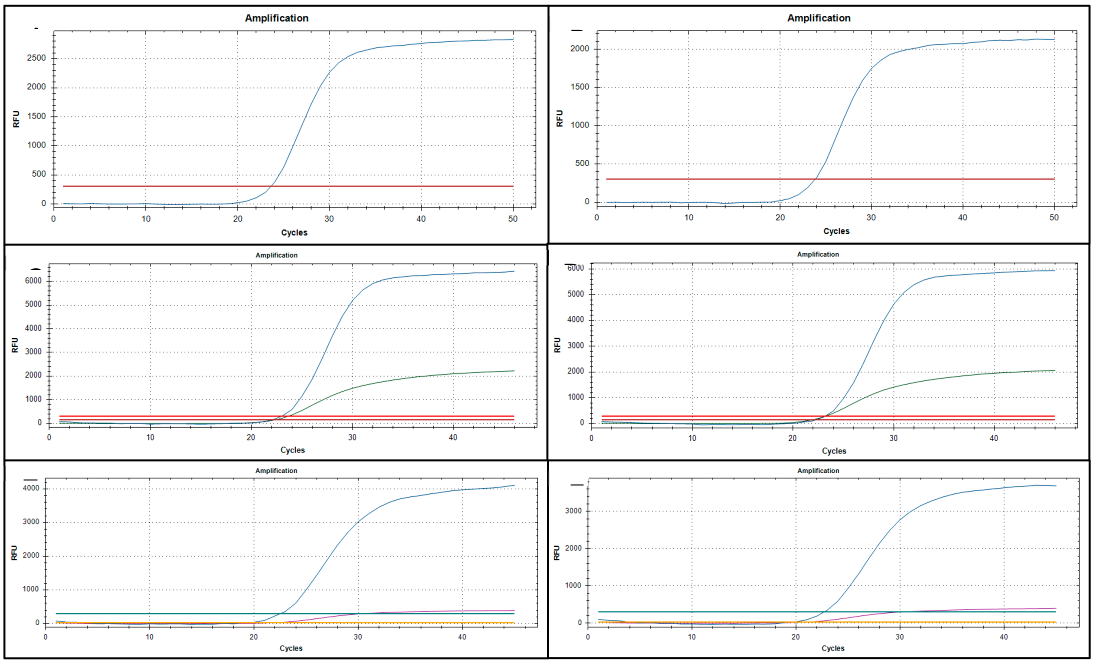

Supplementary Figure S1. Real-time amplification curves of two blood samples, generated by the two commercial kits and the reference protocol, evaluated for the detection of *Leptospira* spp. (A & B: Amplification curves for the lipL32 gene, from the Genesig commercial kit; C & D: Amplification curves for the lipL32 gene (blue line) and endogenous control (red line), from the Viasure commercial kit; E & F: Amplification curves for the lipL32 gene (blue line) and β-actin gene (purple line), from the Duplex 1 reference protocol. Software used for visualization and analysis: CFX Maestro Software, version 2.3
